# Therapeutic potential of epigallocatechin gallate in gynecologic cancer, endometriosis, polycystic ovary syndrome: a mechanistic and translational perspective

**DOI:** 10.3389/fnut.2025.1746959

**Published:** 2026-01-13

**Authors:** Nazlı Tunca Sanlier, İnci Turkoglu, Koray Gorkem Sacinti, Nevin Sanlier

**Affiliations:** 1 Department of Obstetrics and Gynecology, Niğde Çiflik State Hospital, Niğde, Türkiye; 2 Department of Nutrition and Dietetics, Hacettepe University Faculty of Health Sciences, Ankara, Türkiye; 3 Department of Obstetrics and Gynecology, Yale School of Medicine, New Haven, CT, United States; 4 Department of Epidemiology, Department of Public Health, Hacettepe University Faculty of Medicine, Ankara, Türkiye; 5 Department of Nutrition and Dietetics, School of Health Sciences, Ankara Medipol University, Altındağ, Ankara, Türkiye

**Keywords:** endometriosis, epigallocatechin gallate, gynecologic cancer, mechanism of action, polycystic ovary syndrome, signaling pathways

## Abstract

Tea, among the most widely consumed beverages worldwide, is rich in polyphenolic compounds known as catechins, particularly epigallocatechin gallate (EGCG). This review aims to synthesize recent findings and ongoing controversies concerning the role of tea-derived catechins in gynecologic diseases while also outlining key priorities for future research to address existing knowledge gaps. A comprehensive literature search was conducted across the following electronic databases: PubMed, Medline, Embase, Cochrane Library, CINAHL, Web of Science, Scopus, Google Scholar, and ScienceDirect. Epigallocatechin gallate molecules exhibit diverse biological activities, including antioxidant, anti-inflammatory, antiproliferative, and epigenetic effects, primarily through the modulation of key cellular pathways such as PI3K/Akt, MAPK, and NF-κB. Growing evidence from *in vitro, in vivo*, and limited clinical studies suggests that catechins may be of therapeutic value in the treatment of gynecological conditions, including endometrial, ovarian, cervical, and vulvar cancers, as well as non-neoplastic disorders such as polycystic ovary syndrome and endometriosis. EGCG, as both an antioxidant and a pro-oxidant, has been shown to sensitize cancer cells to chemotherapy, regulate hormonal imbalances, and suppress inflammatory responses. However, discrepancies in findings between studies, largely due to heterogeneity in dosage, bioavailability, and study design, limit definitive conclusions. While promising, these compounds require validation through robust, large-scale, and standardized clinical trials to define optimal dosing strategies, assess long-term safety, and determine their roles in routine gynecological practice.

## 1 Introduction

Tea, derived from the leaves of the *Camellia sinensis* plant, is one of the most widely consumed beverages globally (1, 2). Based on the degree of fermentation, tea is classified into six major types: green (unfermented), yellow (lightly fermented), white (lightly fermented), oolong (partially fermented), black (fully fermented) and dark tea (post-fermented) (3). It is estimated that tea contains over 4,000 chemical constituents, among which antioxidant, anticarcinogenic, and antiatherosclerotic properties have been well documented (4).

Flavonoids are considered the principal bioactive compounds responsible for these health effects, with flavanols and flavonols representing the dominant subclasses. Among them, catechins form the most structurally complex and biologically active group (5). During the fermentation process, catechins undergo oxidative conversion to yield dimeric and oligomeric derivatives such as theaflavins, theacitrins, theacinensins, theanaptoquinones, and thearubigins. Compared to unfermented green and white teas, semi-fermented oolong and fully fermented black teas contain fewer catechins but are richer in theaflavins and thearubigins. Catechins, particularly those abundant in fresh tea leaves and green tea, are considered central to tea’s health-promoting attributes (6–8). The biological effects of epigallocatechin gallate (EGCG), the most abundant and extensively studied catechin found in tea, are mediated through pathways involved in apoptosis, cell-cycle regulation, angiogenesis, inflammation and epigenetic modulation in gynecological diseases. These include the induction of apoptosis, cell cycle arrest, anti-angiogenic effects, anti-inflammatory actions, epigenetic modulation, and hormonal regulation. Key signaling pathways affected by EGCG include phosphoinositide 3-kinase (PI3K)/protein kinase B (Akt)/mammalian target of rapamycin (mTOR), mitogen-activated protein kinase (MAPK)/extracellular signal-regulated kinase (ERK), Wnt/*β*-catenin, nuclear factor kappa-B (NF-κB), janus kinase (JAK)/signal transducer and activator of transcription (STAT), hypoxia-inducible factor-1alpha (HIF-1α)/vascular endothelial growth factor (VEGF), and reactive oxygen species (ROS) mediated pathways (9–12). The relationship between catechins and gynecologic diseases is multifaceted and complex, encompassing both potential therapeutic benefits and safety considerations. While mechanistic insights have been progressively uncovered, further research is warranted to clarify their translational relevance. Current evidence is largely derived from *in vitro* experiments, cell lines, animal models, and studies using various tea extracts. This heterogeneity in experimental design and model systems complicates the extrapolation of results to clinical practice and limits definitive conclusions regarding efficacy and safety.

This review aims to synthesize recent findings and ongoing controversies concerning the role of tea-derived catechins in gynecologic diseases, while also outlining key priorities for future research to address existing knowledge gaps and promote evidence-based application.

## 2 Materials and methods

A comprehensive literature search was conducted across the following electronic databases: Medline, PubMed, Embase, Cochrane Library, CINAHL, Web of Science, Scopus, Google Scholar, and ScienceDirect. The search terms included combinations of keywords related to tea and its bioactive compounds “green tea,” “black tea,” “white tea,” “oolong tea,” “flavonoids,” “flavonols,” “epicatechin,” “epigallocatechin,” “epicatechin gallate,” “epigallocatechin gallate,” “antioxidant” as well as gynecologic health conditions “vulvar cancer,” “ovarian cancer,” “cervical cancer,” “endometrial cancer,” “endometriosis,” “polycystic ovary syndrome and “safe intake level.” No date or study-type restrictions were applied during the initial search phase.

### 2.1 Study selection and eligibility criteria

Following deduplication, all titles and abstracts were screened independently by two reviewers to assess relevance. Articles were excluded if they were non-English, preprints, or if the content was deemed irrelevant based on title or abstract. Additional eligible studies were identified through manual screening of reference lists of included articles. Studies were included if they (1) were published between January 2017 and May 2025, (2) were available in full text in English, and (3) addressed the relationship between tea constituents and gynecologic outcomes. Eligible publication types encompassed original research articles, meta-analyses, systematic, narrative, scoping, integrative, umbrella, and traditional reviews. Both *in vitro* and *in vivo* studies, including cell-based, animal, and human models, were considered.

### 2.2 Data extraction and synthesis

Relevant data regarding study design, population or model type, type of tea or compound studied, and gynecologic outcome measures were extracted and narratively synthesized to present an overview of current evidence. Particular attention was given to mechanistic insights involving oxidative stress, inflammation, and hormone regulation.

**Figure 1:**
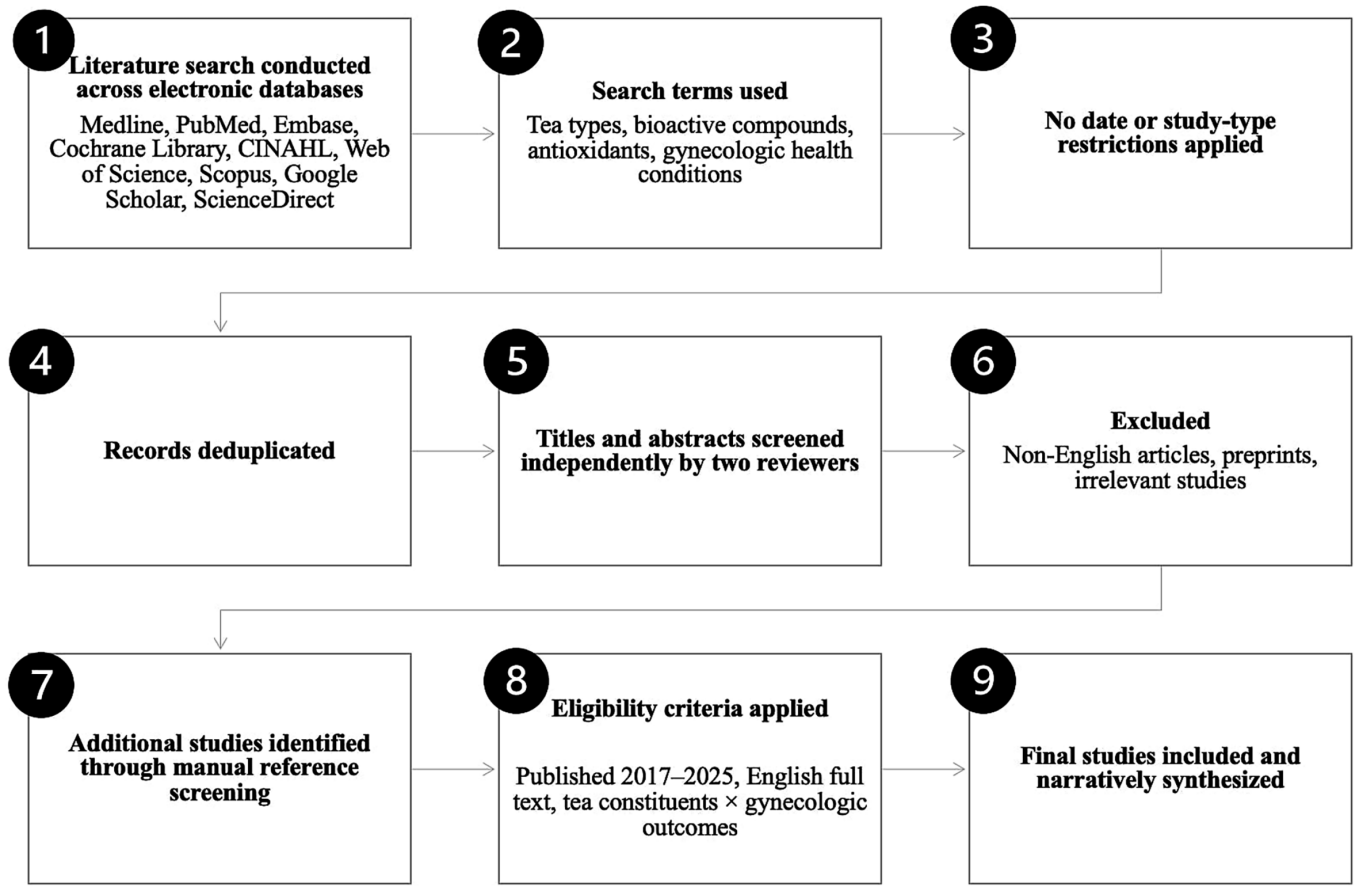
Literature search flow diagram.

The literature search and study selection process is illustrated in Figure 1.

## 3 Phytochemical profile, nutritional composition, bioactivities of tea catechins

Tea is a complex beverage composed of various bioactive substances, including alkaloids, polyphenols, flavonoids, volatile compounds, vitamins, minerals, amino acids, proteins, polysaccharides, lignin, and organic acids. Among the major polyphenolic constituents are catechins, epicatechins, theaflavins, and flavonol glycosides, alongside L-theanine, volatile organic compounds and methylxanthines (13, 14).

Tea is particularly rich in phenolic and flavonoid compounds such as caffeic, chlorogenic, coumaric, ellagic, and gallic acids, as well as kaempferol, myricetin, quercetin, quinic acid, and rutin (15). There exist 8 different catechins in tea: catechin (C), epicatechin (EC), epicatechin gallate (ECG), epigallocatechin (EGC), EGCG, gallocatechin (GC), catechin gallate (CG) and gallocatechin gallate (GCG). Among these, EC, EGC, ECG and EGCG are considered as the predominant catechins. In fact, green tea is a richer resource for catechins, and the most abundant catechin is known as EGCG (4, 16–18). Chemical formulas and some food sources of catechins and energy, nutrient, and phytochemical components of white, green, yellow, oolong, black, and dark teas are shown in Table 1 (19–22), and Table 2 (20).

**Table 1:** Chemical formulas and some food sources of catechins (105–107).

Class/Sub class	Compounds	Food(s)
Catechins (+) - catechin (C)	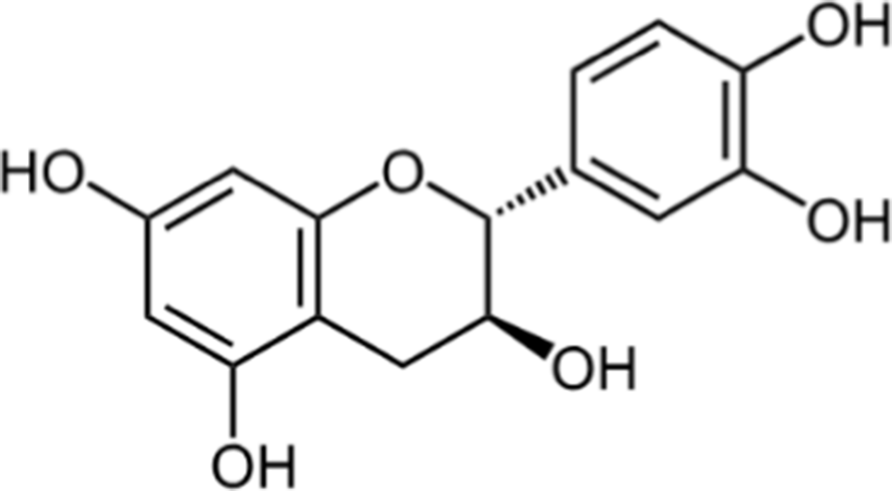	Green tea Black tea Oolong tea Red wine Black Grapes Blackberry Raspberry Chocolate
Catechins (−) - epicatechin (EC)	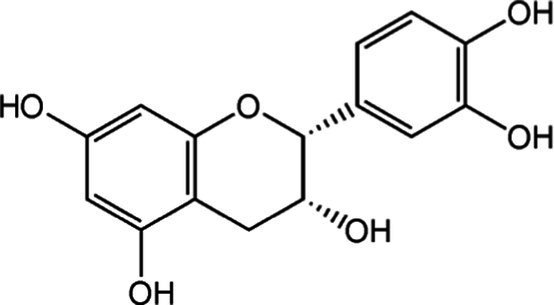	Green tea Red wine Cocoa Apple Blackberry Rosehip
Catechins (−) - epicatechin 3-gallate (ECG)	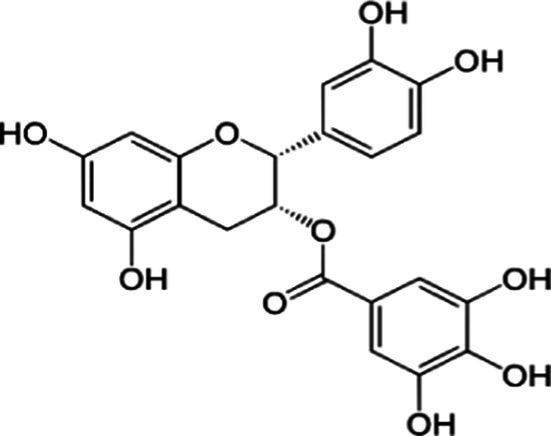	Green tea Black tea Oolong tea Herbal tea Red wine
Catechins (−) - epigallocatechin (EGC)	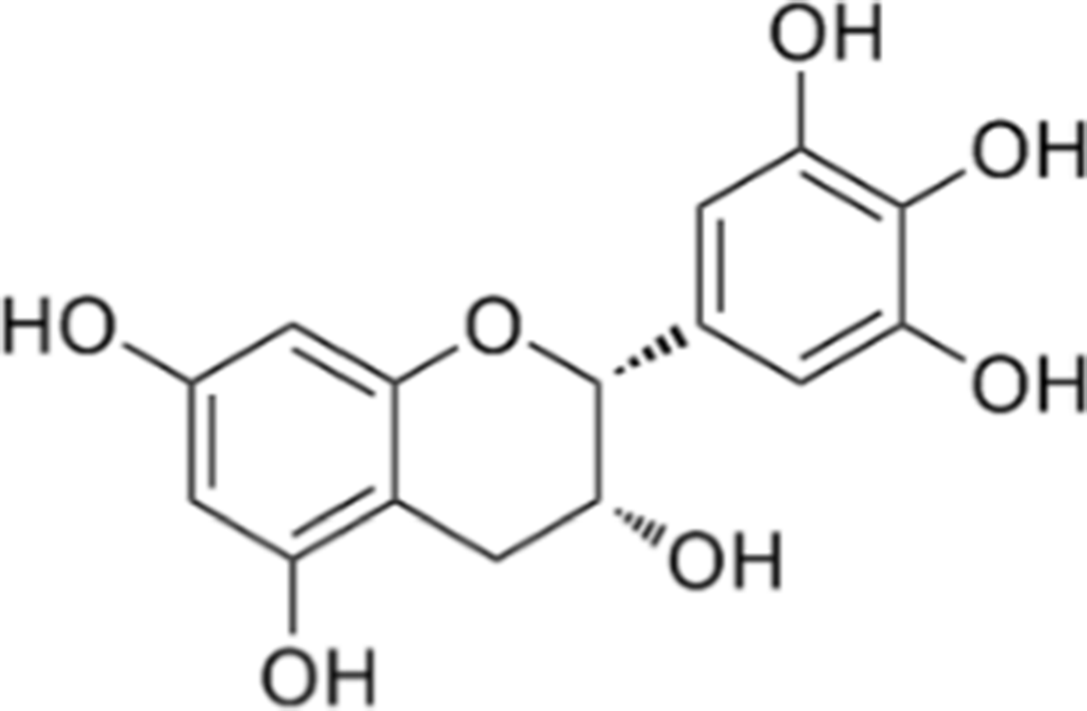	Green tea Black tea Oolong tea
Catechins (−) - epigallocatechin 3-gallate (EGCG)	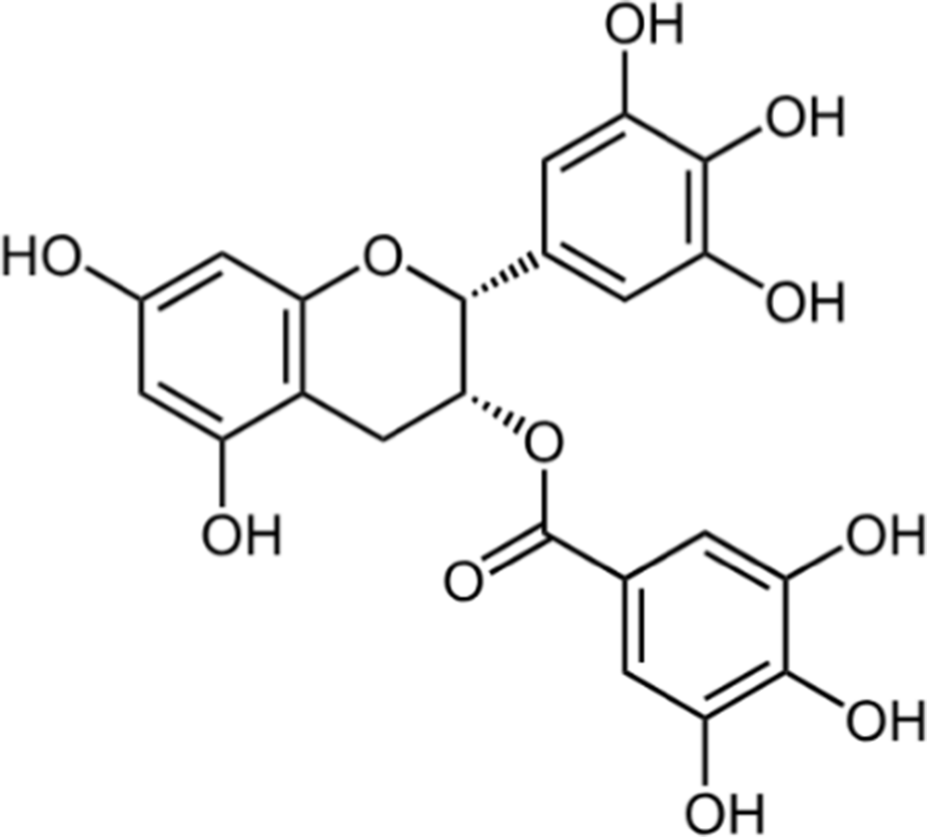	Green tea Black tea Oolong tea Herbal tea Red wine
Catechins (−) - catechin gallate (CG)	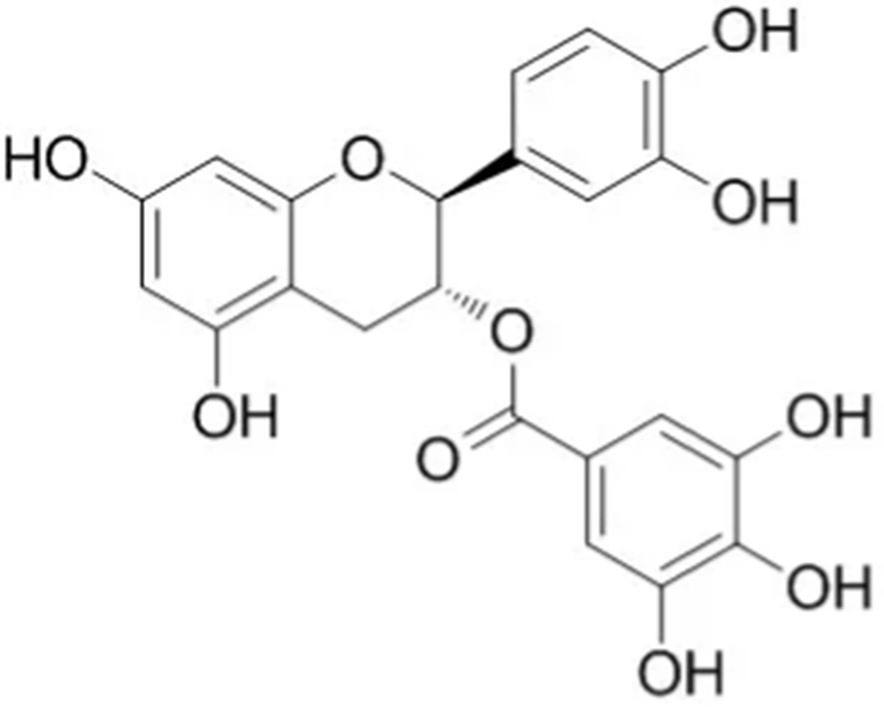	Green tea Black tea Oolong tea Herbal tea
Catechins (−) - gallocatechin (GC)	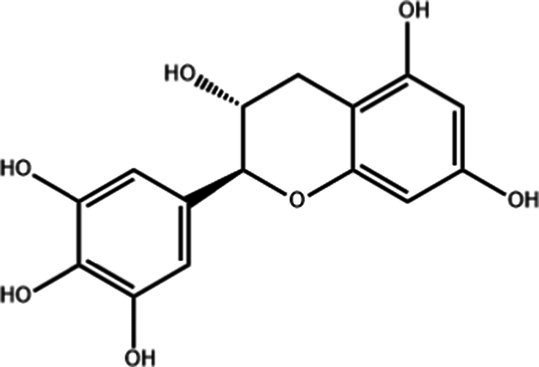	Herbal tea Red wine
Catechins (−) - gallocatechin gallate (GCG)	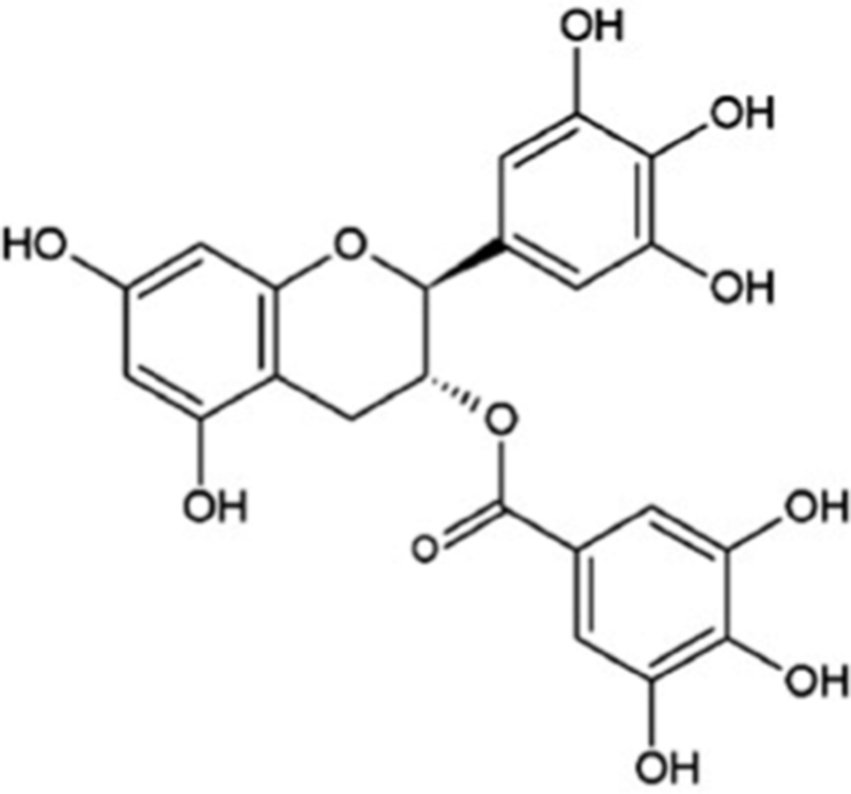	Green tea Black tea Oolong tea Herbal tea

**Table 2:** Phytochemical content (mg/g DW) of white, green, yellow, oolong, black and dark tea (20).

Phytochemicals	White tea	Green tea	Yellow tea	Oolong tea	Black tea	Dark tea
Catechin	ND	1.37	1.32	ND	ND	4.93
EC	ND	6.20	5.97	1.58	0.74	10.36
GC	ND	2.74	1.86	2.51	ND	5.54
EGC	8.42	13.66	13.09	31.25	ND	23.43
Cg	ND	0.35	ND	ND	ND	ND
ECG	3.14	30.49	35.40	8.44	3.51	10.88
GCG	ND	1.45	ND	ND	0.51	0.93
EGCG	6.01	50.78	59.35	36.70	3.80	10.89
Gallic acid	2.18	0.94	1.43	3.28	3.55	3.10
Chlorogenic acid	ND	ND	0.37	ND	0.19	0.28
Ellagic acid	ND	1.88	2.14	1.88	2.61	2.21
Kaempferol-3-G	0.50	1.05	1.61	1.19	1.45	1.00
Theaflavin	ND	ND	ND	ND	0.56	0.48
Caffeine	27.47	41.46	39.76	34.77	41.63	27.08

CG, Catechin gallate; DW, Dry weight; EC, Epicatechin; ECG, Epicatechin gallate; EGC, Epigallocatechin; EGCG, Epigallocatechin gallate; GC, Gallocatechin; GCG, Gallocatechin gallate; ND, Not detected. Adapted from (20), licensed under CC BY 4.0.

## 4 The effect of tea catechins on gynecological diseases

The function of tea for gynecological diseases is complex and multifaceted. Additionally key tea constituents such as EGCG and theaflavin, have attracted considerable attention for their therapeutic potential (11, 23). In this context, it is worth stating that tea represents major dietary sources of daily polyphenol intake (24). The influence of catechins, particularly EGCG, in gynecological disorders are mediated through antioxidant, anti-inflammatory, anti-proliferative, and apoptotic mechanisms as well as epigenetic regulation. EGCG, a potent antioxidant that mitigates oxidative stress via neutralizing ROS, is especially relevant due to the established link between oxidative stress and ovarian dysfunction (25, 26).

**Figure 2:**
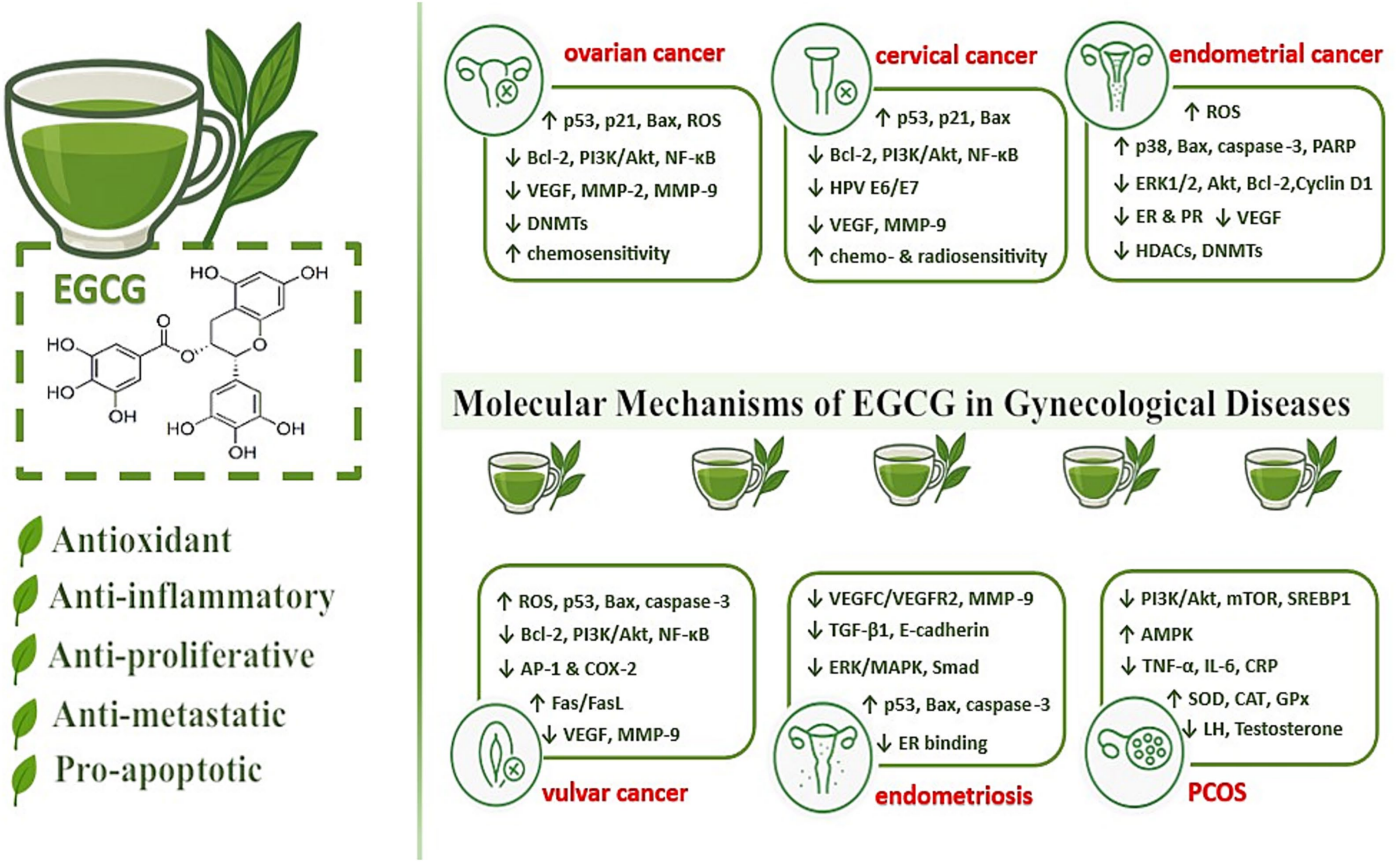
Molecular mechanisms of EGCG in gynecological diseases. This figure summarizes the principal molecular mechanisms by which EGCG exerts its beneficial effects in gynecological diseases, including ovarian, cervical, endometrial, and vulvar cancers, endometriosis, and PCOS. EGCG demonstrates antioxidant, anti-inflammatory, anti-proliferative, anti-metastatic, and pro-apoptotic activities by modulating key signaling pathways, reducing pro-tumorigenic factors, and enhancing sensitivity to chemo- and radiotherapy. The figure highlights the potential of EGCG as a multi-targeted adjunct in the management of various gynecological conditions. AP-1, activator protein-1; Bax, Bcl-2-associated X protein; Bcl-2, B-cell lymphoma 2; CAT, catalase; COX-2, cyclooxygenase-2; CRP, C-reactive protein; DNMTs, DNA methyltransferases; E-cadherin, epithelial cadherin; EGCG, epigallocatechin-3-gallate; ER, estrogen receptor; GPx, glutathione peroxidase; HDACs, histone deacetylases; HPV, human papillomavirus; IL-6, interleukin-6; LH, luteinizing hormone; MAPK, mitogen-activated protein kinase; MMP, matrix metalloproteinase; mTOR, mammalian target of rapamycin; NF-κB, nuclear factor kappa-B; PARP, poly (ADP-ribose) polymerase; PCOS, polycystic ovary syndrome; PI3K, phosphoinositide 3-kinase; PR, progesterone receptor; ROS, reactive oxygen species; SOD, superoxide dismutase; SREBP1, sterol regulatory element-binding protein 1; TGF-β1, transforming growth factor-beta 1; TNF-α, tumor necrosis factor-alpha; VEGF, vascular endothelial growth factor; VEGFR, vascular endothelial growth factor receptor.

Catechin treatment significantly reduces tumor necrosis factor-*α* (TNF-α), interleukin-1β (IL-1β), and interleukin-6 (IL-6) levels, while also alleviating inflammatory and oxidative damage by enhancing the activity of antioxidant enzymes such as superoxide dismutase (SOD), catalase (CAT), and glutathione peroxidase (GPX) in ovarian tissue (27, 28). Catechins inhibit proliferation by interfering with growth factor-mediated signaling pathways, including receptor tyrosine kinases (RTKs) such as VEGF, which carries a prominent role in the pathogenesis of proliferative gynecological diseases. EGCG impacts epigenetic mechanisms which may shift gene expression without altering the deoxyribonucleic acid (DNA) sequence. This involves modulation of DNA methylation as well as histone modification, which are crucial for the development and progression of gynecological diseases (29). Molecular mechanisms of EGCG in gynecological diseases are shown in Figure 2.

## 5 Tea catechins and anticancer effects

Tea and its ingredients, particularly EGCG, are known to exert antimutagenic effects by interacting with carcinogenic compounds and neutralizing ROS prior to the initiation of DNA damage. Catechins have been reported to protect cellular membranes from oxidative damage and to inhibit membrane-bound receptors that are essential for cancer cell proliferation. Tea polyphenols are known to suppress angiogenesis, metastasis, and cell proliferation, which are crucial processes promoting tumor growth (30–33). The antitumor effects of polyphenols are primarily mediated through the inhibition of cell proliferation and the induction of phase II antioxidant enzymes, including SOD, glutathione s-transferase (GST), GPX, and glutathione reductase (GR). It is recorded that consuming four cups of green tea daily for 4 months significantly reduces urinary levels of 8-hydroxydeoxyguanosine, a biomarker of oxidative DNA damage (34).

The anticancer impact of EGCG has been attributed to its ability to inhibit osteoblastic differentiation (35). Furthermore, cancer stem cells are employed to spot the potential anticancer impacts of EGCG (36). Stem cells (precursor cells) possess the capacity to proliferate, self-renew, and maintain a stable cell population, while also retaining the ability to differentiate into specific cell types when required. Both green tea extract and EGCG are the agents that limit cell growth in these cellular and animal models (37). *In vivo* and *in vitro* research suggest that cancer stem cells are key drivers of tumor recurrence and metastatic progression. Recent evidence indicates that cancer stem cells possess an enhanced oncogenic potential compared to non-stem cancer cells and play a critical role in facilitating epithelial mesenchymal transition (EMT) during metastasis, thereby enabling tumor cells to intravasate into the vasculature (36). It is announced that EGCG can demonstrate multiple bioactivities, particularly anticancer impacts, via inhibiting cancer stem cells and regulating molecular events ascribed to cancer cell proliferation, apoptosis, immunity, and others (38). EGCG can induce apoptosis in several cancer models, in particular intrinsic and extrinsic apoptotic pathways. The major pathways deployed in the process of controlling cell proliferation are cell cycle arrest and induction of apoptosis. Catechols present in green tea is able to regulate both the G1/S transition and the G2/M transition, along with preventing the boost in cell number and DNA synthesis (39).

It is merely one of the biological reactions regulated by NF-κB in the process of cell proliferation and cancer cell death. Moreover, green tea catechins particularly EGCG stimulate eNOS activity (40). It is recorded that EGCG inhibits MAPKs including like ERK, jun n-terminal kinases (JNK), and p38 and helps numerous pathophysiological processes, involving cell proliferation, differentiation, and apoptosis in cancer cells (41). Also, studies have reported that exposure to EGCG inhibits TNF-*α* activity, thereby promoting cancer cell death (35). Green tea polyphenols may exert anticancer effect by modulating DNA methylation, micro ribonucleic acid (RNA), and histones (42). By reducing the constitutive activation of STAT3 and NF-κB in cancer cells, EGCG suppress VEGF production. EGCG addresses the PI3K/Akt pathway and PI3K. EGCG is known to trigger apoptosis via PI3K/Akt, in spite of the fact that the molecular mechanisms behind the combined action of autophagy and apoptosis still remain unclear (43). In the 5,637 cell line, both EGCG and the PI3K/Akt inhibitor LY294002 induced apoptosis; However, their combined application resulted in the highest apoptotic efficacy (44). EGCG is reported to inhibit cell proliferation by inactivating the PI3K/Akt pathway and facilitating the interaction between autophagy and apoptosis. Similarly, EGCG administration reduced Akt phosphorylation in PANC-1 cells, suggested that downregulation of p-Akt is linked with the development of cytotoxic autophagy (45).

In summary, while catechins are promising in the management of gynecologic diseases, their clinical applicability remains limited. The majority of supporting evidence is derived from *in vitro* studies and animal models, while robust, well-designed clinical trials in humans are still lacking. Additionally, the effects of catechins may vary depending on molecular parameters such as VEGF levels, which can differentially influence angiogenesis and inflammation (28). Thence, prospective research, particularly human clinical trials is essential to establish the efficacy and safety of catechins in the treatment of gynecological conditions.

## 6 The relationship between gynecologic cancers and EGCG

The effects of EGCG on cancer cells are mediated through a number of cellular signaling pathways and mechanisms. These cover mitogen-activated protein (MAP) kinases and activator protein 1 (AP-1), NF-κB signaling pathways, epidermal growth factor receptor (EGFR)-mediated signaling pathways, and insulin-like growth factor 1 (IGF-1)-mediated signal transduction pathways. EGCG is known to inhibit cancer cell proliferation via interfering with cell cycle progression and inducing apoptosis. These effects are mediated through the regulation of varying signaling pathways such as NF-κB inhibition and nuclear factor erythroid 2-related factor 2 (Nrf2) activation (46, 47). EGCG has also been shown to reduce Tumor cell invasion and metastasis by inhibiting proteasome activity, controlling matrix metalloproteinase (MMP) activity, and suppressing urokinase-plasminogen activator activity.

In addition, EGCG has been reported to suppress cancer cell proliferation and inhibit tumor progression by inducing apoptosis and arresting the cell cycle (48). EGCG modulates epigenetic mechanisms, by regulating gene expression and inhibiting key enzymes involved in cancer progression, such as DNA DNMTs and histone deacetylases (HDACs) (29, 46). By scavenging free radicals, catechins decrease oxidative stress, which is known as contributor to cancer development and progression (10, 47).

### 6.1 The relationship between endometrial cancer and EGCG

There are limited studies investigating the link between endometrial cancer and catechin consumption. The evaluations of various tea types have revealed a significant inverse relationship between the consumption of black and green tea and the risk of endometrial cancer (49). EGCG can inhibit tumor growth by blocking cell proliferation, inducing apoptosis, and suppressing angiogenesis in endometrial cancer models. EGCG has attracted significant attention for its ability to modulate various cellular pathways and its potential as an adjuvant therapy in endometrial cancer.

#### 6.1.1 Mechanisms of EGCG in endometrial cancer

EGCG exhibits anti-angiogenic properties by inhibiting VEGF production, an eminent component in tumor angiogenesis. This impact was observed in endometrial cancer cell cultures, where EGCG significantly reduced VEGF levels (29). EMT, a key process in cancer metastasis, is inhibited, thereby reducing the dissemination of cancer cells (50).

EGCG inhibits the proliferation of endometrial cancer cells and induces apoptosis. It down-regulates proliferation markers such as estrogen receptor *α*, progesterone receptor, and cyclin D1, while up-regulating pro-apoptotic proteins such as Bax and down-regulating anti-apoptotic proteins such as Bcl2. This leads to the activation of caspase-3 and poly (ADP-ribose) polymerase, which are hallmarks of apoptosis. In addition, EGCG has been shown to inhibit the proliferation of endometrial cancer cells by causing cell cycle arrest in the G0/G1 phase, an effect mediated through the downregulation of estrogen and progesterone receptors, which play critical roles in tumor development (7, 46, 51).

EGCG initiates ROS formation, which plays a critical role in its pro-apoptotic effects. Increased ROS levels lead to oxidative stress and contribute to the activation of the p38 MAP kinase pathway, which is crucial for EGCG-induced apoptosis in endometrial cancer cells (50). EGCG affects epigenetic modifications by interacting with DNMTs and HDACs this interaction can lead to changes in gene expression that suppress cancer cell growth and promote apoptosis (46).

The precursor of EGCG, pro-epigallocatechin gallate (pro-EGCG), reduces the formation of new blood vessels in endometrial cancer by down-regulating vascular endothelial growth factor A (VEGFA), HIF-1α and C-X-C motif chemokine ligand 12 (CXCL12), which is achieved by inhibiting the PI3K/Akt/mTOR/HIF-1α pathway (52). Pro-EGCG has been found to exhibit superior anti-cancer activity in endometrial cancer models (50). However, it is thought that these findings may be the result of pharmacological potentiation of EGCG rather than evidence of a separate therapeutic agent. The purpose of discussing Pro-EGCG in this section is to contextualize the mechanisms of EGCG, given that Pro-EGCG was developed to address the low bioavailability and stability issues associated with natural EGCG.

The effects and mechanism/roadmap of EGCG on endometrial cancer are shown in Table 3.

**Table 3:** EGCG effects and mechanism/pathway on endometrial, ovarian and cervical cancer cells.

Effect	Mechanism/Pathwat Involved	Refs.
Inhibits angiogenesis	Downregulates VEGFA, HIF-1α, CXCL12	(50, 51)
Reduces tumor cell proliferation	Cell cycle arrest, MAPK/Akt inhibition	(46, 51, 83)
Induces apoptosis	Caspase activation, Bcl-2/Bax modulation	(7, 46, 52)
Downregulates hormone receptors	Decreases estrogen/progesterone receptor expression	(108)

Protein/Pathway	EGCG effects on ovarian cancer cells	Refs.
JUN, FADD, NFKB1	Regulation of cell apoptosis	(63)
Bcl-2, Bax, Bcl-X (L)	Modulation of apoptosis	(63)
HIF-1α	Potential regulation of hypoxia response	(63)
MMP2	Inhibition of migration/invasion	(57)
NF-κB, S100A4	Downregulation, reduced proliferation	(63)

Mechanism/Pathway	EGCG effects in cervical cancer cells (65, 70–72, 74)
TGF-*β*/ROS/Smad signaling	Inhibits EMT, reduces invasion/metastasis
Epigenetic enzyme modulation	Reactivates tumor suppressors, suppresses oncogenes
Angiogenesis signaling	Reduces pro-angiogenic gene expression
EGF/EGFR pathway	Blocks migration, ECM remodeling

Akt, Protein kinase B; Bax, Bcl-2–associated X protein; Bcl-2, B-cell lymphoma 2; Bcl-X (L), B-cell lymphoma-extra large; CXCL12, C-X-C motif chemokine ligand 12; ECM, Extracellular matrix; EGF, Epidermal growth factor; EGFR, Epidermal growth factor receptor; EMT, Epithelial mesenchymal transition; FADD, Fas-associated death domain; HIF-1α, Hypoxia-inducible factor-1alpha; JUN, Jun proto-oncogene; MAPK, Mitogen-activated protein kinase; MMP-2, Matrix metalloproteinase-2; NF-κB, Nuclear factor kappa-B; NFKB1, Nuclear factor kappa B subunit 1; ROS, Reactive oxygen species; TGF-β, Transforming growth factor beta; VEGFA, Vascular endothelial growth factor A.

#### 6.1.2 Pathways involved in the effect of EGCG

EGCG affects the MAPK and Akt signaling pathways. It phosphides ERK and Akt, which are critical mediators of cell survival and proliferation. EGCG affects various signaling pathways including inhibition of MAPK and Akt pathways which are important for both cell survival and proliferation and activates p38 MAP kinase which is functional in promoting apoptosis. EGCG is also known to modulate epigenetic factors by interacting with DNA methyltransferases and histone deacetylases which may alter gene expression patterns in cancer cells (53). It promotes apoptosis by inhibiting the phosphorylation of Akt (51).

EGCG increases the phosphorylation of JNK and p38 MAPK, which are involved in the stress response and apoptosis. This activation is associated with increased apoptotic activity in endometrial cancer cells (51). EGCG inhibits the STAT3 signaling pathway, which is known to promote cell survival and proliferation. By impairing STAT3 activity, EGCG reduces the expression of genes involved in cell growth and survival, such as B-cell lymphoma-extra large (Bcl-xL) and cyclin D1 (51, 53). Pro-EGCG, a derivative of EGCG, was developed to overcome the limitations of EGCG’s poor bioavailability. Pro-EGCG exhibits significant antiproliferative and pro-apoptotic effects in endometrial cancer models, exhibiting improved stability and efficacy. It exhibits superior anticancer activity compared to EGCG by effectively inhibiting tumor growth and angiogenesis (51, 53). Even though EGCG demonstrates notable anticancer potential, it is deemed important to reconsider the broader context of catechins in cancer prevention. Nevertheless, the relevant data displayed mixed results about the protective effects of catechins against some cancers, potentially due to variations in their bioavailability and interactions with other dietary components (47). EGCG holds significant potential as an adjuvant therapy in endometrial cancer due to its ability to target multiple molecular pathways involved in tumor progression. Nevertheless, this might pose some challenges, like its poor bioavailability and the need for relatively high doses to achieve therapeutic efficacy (51).

There exists a limited number of studies inquiring into the link between endometrial cancer and the consumption of catechins. Evidence from *in vitro, in vivo*, and clinical studies indicates potential therapeutic benefits of these compounds, including enhanced chemotherapeutic efficacy and regulation of inflammatory and hormonal imbalances. However, findings remain inconsistent due to variability in dosage, bioavailability, and study design. Despite promising results, robust, large-scale, and standardized clinical trials are urgently needed to validate efficacy, determine optimal dosing, and assess long-term safety. Integrating dietary polyphenols into evidence-based gynecological care may offer novel preventive and therapeutic strategies, provided individual variability and disease heterogeneity are accounted for. Due to methodological variability across existing studies, it remains challenging to establish a comprehensive assertion that tea consumption offers unequivocal protection against endometrial cancer. However, due to EGCG’s poor bioavailability and the requirement for higher doses to achieve therapeutic efficacy, along with the current lack of comprehensive data on its safety and effectiveness in the treatment of endometrial cancer, well-designed clinical trials are still needed.

### 6.2 Relationship between ovarian cancer and EGCG

A meta-analysis showed a possible inverse link between green tea consumption and ovarian cancer risk (54). Ovarian cancer risk parameters encompass nulliparity, early menstruation, obesity and family history of breast or ovarian cancer (55). Green tea, rich in the bioactive compound EGCG, exhibits notable anti-cancer properties. Evidence suggests that green tea consumption is associated with a reduced risk of ovarian cancer progression. EGCG exerts antiproliferative, antiangiogenic, and antimetastatic effects, while also promoting apoptosis and autophagy in ovarian cancer cells (46).

#### 6.2.1 Mechanisms and pathways of EGCG in ovarian cancer

Ovarian cancer tumorigenesis may be due to dysregulations in the PI3K/Akt/mTOR signaling pathway. Mutations or amplifications in the PIK3CA gene, mutations in the phosphoinositide-3-kinase regulatory subunit (PIK3R) gene, loss of phosphatase and tensin homolog (PTEN), and mutations or amplifications in AKT isoforms result in uncontrolled activation of this signaling pathway and result in the development of ovarian cancer (56). EGCG upregulates PTEN expression and downregulates PDK1, phospho-Akt, and phospho-mTOR, leading to inhibition of ovarian cancer cell proliferation and induction of apoptosis. This effect is reversed by PTEN inhibitors, highlighting the critical role of the PTEN/Akt/mTOR pathway in mediating the anticancer effects of EGCG. Inhibition of the Akt/mTOR pathway is known to induce apoptosis of cancer cells (57).

In platinum-resistant ovarian cancer, EGCG up-regulates p53 and down-regulates S100A4 and NF-κB, resulting in decreased cell proliferation and increased apoptosis. This suggests that EGCG may overcome drug resistance by targeting the S100A4/NF-κB axis (58). EGCG reduces cell proliferation and invasion by inhibiting the endothelin A receptor (ET(A)R)/endothelin-1 (ET-1) axis. It also reduces the activation of p42/p44 and p38 MAPK pathways that play a role in cell growth and angiogenesis, thereby inhibiting tumor growth and progression (59, 60). EGCG prevents the acquisition of cancer stem cell phenotypes in ovarian cancer tumorspheres by inhibiting Src and JAK/STAT3 signaling pathways. This action reduces the expression of cancer stem cell markers and inhibits cell chemotaxis, suggesting a role in preventing metastasis and tumor recurrence (61). The VEGF pathway down-regulates VEGF expression, reduces angiogenesis, and tumor growth via the EGCG, Transforming growth factor beta 1 (TGF-β1)/mothers against decapentaplegic (SMAD), and 67-kDa laminin receptor-mediated cAMP response element-binding protein (CREB) pathways. This mechanism is particularly important in alleviating ovarian hyperstimulation syndrome, a condition characterized by increased VEGF levels (62). EGCG affects other signaling proteins such as jun proto-oncogene (JUN), fas-associated death domain (FADD), nuclear factor kappa B subunit 1 (NFKB1), B-cell lymphoma 2 (Bcl-2), HIF-1α, and MMP, which are involved in cell cycle regulation, DNA replication, and cellular organization. It also inhibits cell proliferation and migration in ovarian cancer cells by modulating p38 kinase and matrix metalloproteinase-2 (MMP-2) (57, 63). Although EGCG shows significant potential in targeting various pathways in ovarian cancer, it is important to consider the complexity of cancer biology and the potential for the development of resistance mechanisms. In addition, the bioavailability and pharmacokinetics of EGCG in humans require further investigation to optimize its therapeutic efficacy. Future research should focus on clinical studies to confirm these findings and explore the potential of EGCG as a complementary therapy in the treatment of ovarian cancer. The effect of EGCG on ovarian cancer cells is presented in Table 3.

The proapoptotic aspects of EGCG are attributed to the upregulation of proapoptotic proteins p21, Bax and caspase-3 and reduced expression of antiapoptotic proteins proliferating cell nuclear antigen (PCNA), Bcl-2 and Bcl-Xl (57). It is reported that EGCG treatment led to ovarian cancer regression in female athymic mice. This anticancer effect is believed to occur through the modulation of multiple cellular signaling pathways, particularly the PI3K/Akt/mTOR pathway, which is critically involved in uncontrolled cell proliferation and metastasis. Dysregulated autophagy is known to have a role in the pathogenesis of ovarian cancer, and disruption of autophagy regulators like LC-3BII, p62, autophagy related 5 (ATG5), Beclin1 and unc-51 like kinase 1 (ULK1) induces ovarian cancer via promoting cellular growth and preventing autophagosome formation (64). In another study, green tea extract (25, 50 μg/mL) in combination with a chemotherapy agent that prevents the function of microtubules displayed a synergistic impact on the induction of apoptosis in ovarian cancer cell lines (SKOV-3 and OVCAR-3). The combined treatment was believed to augment the expression of proapoptotic proteins (especially Bax, CytC, caspase-3, and caspase-9) and reduction in the expression of antiapoptotic proteins like Bcl-2 via inhibiting Akt phosphorylation (65). Catechins have been shown to enhance the efficacy of chemotherapeutic agents by modulating drug resistance mechanisms. Pretreatment with catechins increases the cytotoxicity of cisplatin in ovarian cancer cells, promising a potential role in combination therapies (66). Relatively few studies have examined the relationship between tea consumption and survival following ovarian cancer diagnosis. The limited data proves that a potential survival benefit associated with higher green tea intake (67).

Research suggests that tea consumption is associated with a reduced risk of ovarian cancer. It was found out that black tea consumption displayed linear suppressive effects on ovarian cancer risk, and the risk reduced by 30% in persons that consumed ≥2 cups daily, and green tea consumption significantly decreased the risk of ovarian tumor (68).

### 6.3 The relationship between cervical cancer and EGCG

Oncogenic strains of human papillomavirus 16 (HPV16) and human papillomavirus 18 (HPV18) infections have been proven as causal agents for cervical cancer. The other risk elements involve smoking, having a history of multiple sexual partners, unprotected intercourse, and earlier sexual activity. Even though the prevention of cervical cancer is indeed possible with the HPV vaccine, prospective studies are called for the treatment of active disease. EGCG and green tea, known for their anti-cancer properties, possess therapeutic potential in the treatment of cervical cancer (69).

#### 6.3.1 Mechanisms and pathways of EGCG in cervical cancer

Epigallocatechin-3-gallate (EGCG), a major polyphenol found in green tea, has demonstrated multiple anti-cancer effects in cervical cancer through various molecular mechanisms and pathways. EGCG inhibits cervical cancer cell growth, anti-metastasis, anti-proliferation, and angiogenesis by targeting key signaling pathways, regulating epigenetic regulators, and suppressing tumor-promoting processes such as EMT and extracellular matrix remodeling (70).

EGCG suppresses TGF-*β*-induced EMT, a process critical for cancer cell invasion and metastasis, by ROS generation. Inhibiting Smad2/3 phosphorylation, nuclear translocation, DNA binding, and transcriptional activity. Downregulating mesenchymal markers (vimentin, ZEB, slug, snail, and twist) and upregulating E-cadherin, thus reversing EMT in cervical cancer cells. Specifically, EGCG exhibited anti-metastatic properties in HeLa and SiHa cells by inhibiting the epithelial-to-mesenchymal mediated transition, a process mediated by TGF-β through the ROS/Smad signaling pathway (66). The effect of EGCG has been demonstrated by inhibiting the growth of cervical cancer cells such as CaSki and HeLa cells in a time- and concentration-dependent manner, inducing cell cycle arrest, and promoting apoptosis, evidenced by increased caspase-3 activity and morphological changes in cancer cells (29, 68). EGCG reduces the activity of DNMTs and HDACs, leading to attenuation of gene silencing. It reverses promoter hypermethylation and reactivates tumor suppressor genes (TP73, PTEN, SOCS1, CDH1, RARβ, DAPK1). It down-regulates key oncogenic signaling pathways (PI3K, Wnt, MAPK) and cell cycle/metastasis regulators (TERT, CCNB1/2, MMP2/7, IL6) (71). It affects DNA methylation and microRNA dysregulation, which are important in cervical cancer progression (72).

Furthermore, EGCG affects the expression of key oncogenes and tumor suppressor genes. It regulates the expression of HPV oncogenes E6 and E7, which are crucial for cervical cancer progression, apoptosis and genes involved in cell cycle regulation (73). EGCG affects epigenetic mechanisms such as DNA methylation and microRNA regulation, which play a critical role in cervical cancer development. These changes can occur before genetic mutations, making them potential targets for cancer prevention (72). EGCG up-regulates genes with anti-angiogenic effects while down-regulating genes involved in angiogenesis, cell proliferation, adhesion, motility, and invasion. It results in decreased proliferation, adhesion, dissemination, and invasiveness of cervical cancer cells (70). EGCG inhibits epidermal growth factor (EGF)-induced EMT, migration, and extracellular matrix (ECM) remodeling by: reducing MMP-2 expression and activity. Inhibiting EGF-induced colony formation and migration in both HPV-positive and HPV-negative cervical cancer cells (74). EGCG Effects and mechanism/pathway on cervical cancer cells are presented in Table 3.

EGCG exerts its anticancer effects by targeting multiple pathways in cervical cancer: it inhibits EMT and metastasis via ROS/Smad and EGF/EGFR signaling, regulates epigenetic regulators to reactivate tumor suppressor genes, and suppresses angiogenesis and matrix remodeling. These multi-targeted actions suggest the potential of EGCG as a complementary therapeutic agent in the management of cervical cancer.

EGCG down-regulates key signaling pathways involved in cancer cell proliferation and survival, including PI3K, Wnt, and MAPK pathways (71). It also inhibits the HIF-1α and VEGF pathways, which are critical for tumor angiogenesis. EGCG reduces protein synthesis and cell migration by inhibiting the PI3K/Akt/mTOR signaling pathway (72). Although EGCG shows significant promise in the treatment of cervical cancer, it is important to consider the broader implications of its use. The regulation of epigenetic and signaling pathways by EGCG suggests its potential application in other types of cancer. However, the variability in response among different individuals and cancer subtypes requires further investigation to optimize its therapeutic use. In addition, the development of EGCG nanoformulations may increase its bioavailability and therapeutic efficacy (75). Future studies should focus on clinical trials to confirm these findings and explore the long-term effects and safety of EGCG in cancer treatment.

Due to its ability to regulate key pathways involved in cancer progression, EGCG is recognized as an anti-chemotherapy agent for cervical cancer. Its natural origin and low toxicity make it an attractive alternative to conventional chemotherapy, which often has significant side effects. However, challenges such as its bioavailability and the need for standardized formulations must be addressed to maximize its therapeutic potential. The anticancer potential of is by several *in vitro* and *in vivo* studies. Howbeit clinical trials are necessary to be able to speak about their efficacy and safety in human beings. What is more, the variability observed in research findings implies the necessity of prospective studies to fully delve into the relevant mechanisms, establish standardized protocols, and validate their benefits in various patient populations and to optimize clinical application of catechins in cancer therapy (10, 47).

### 6.4 Vulvar cancer and EGCG relationship

Vulvar cancer is a malignant disease that is often associated with human papillomavirus (HPV) infection (76). While the expression of catechins in vulvar cancer offers valuable insights into disease progression and potential recurrence, it is essential to reconsider the multifactorial nature of vulvar cancer and consider it within a broader biological and clinical context (77). Its use as a topical agent for HPV-associated lesions (e.g., VIN) is supported by its ability to inhibit proliferation and promote apoptosis. EGCG exhibits multiple mechanisms of action and signaling pathways that contribute to its anticancer effects, such as modulation of autophagy, inhibition of key signaling pathways, and degradation of viral oncoproteins.

#### 6.4.1 Mechanisms of action and signaling pathways of EGCG in vulvar and HPV-related cancers

EGCG promotes the degradation of HPV E6 and E7 oncoproteins in HPV18-infected keratinocytes and VIN cells, leading to decreased cell proliferation. This degradation is mediated by the proteasome pathway, as shown by the accumulation of E6/E7 proteins when a proteasome inhibitor is added. EGCG does not affect E6/E7 messenger RNA (mRNA) levels but increases protein turnover, resulting in upregulation of tumor suppressor genes (p53, p21, pRb) and inhibition of viral replication (78).

EGCG induces apoptosis by inhibiting cell proliferation in HPV-associated lesions. This is accompanied by downregulation of E6/E7 proteins and activation of tumor suppressors. However, it also suggests that EGCG-mediated E6 degradation may occur through mechanisms other than proteasome-mediated polyubiquitination. EGCG has been shown to regulate autophagy, a cellular degradation process that can affect cancer cell survival and death. By regulating autophagy, EGCG can promote tumor suppression or enhance tumor cell death, depending on the context. In reproductive cancers, including vulvar cancer, the ability of EGCG to balance autophagy may contribute to its therapeutic potential as a stand-alone treatment or in combination with other chemotherapeutic agents (53). In HPV-associated cancers such as vulvar cancer, EGCG has been shown to promote proteasomal degradation of HPV oncoproteins E6 and E7. This degradation leads to reactivation of tumor suppressor pathways and inhibition of viral replication. The ability of EGCG to target these oncoproteins suggests its potential as an antiviral agent that may be particularly useful in HPV-associated vulvar cancer (78).

It has been reported that EGCG increases the efficacy of existing chemotherapeutic agents through synergistic effects, allowing the use of lower doses of these drugs and reducing their associated toxicity. This feature makes EGCG a valuable adjunct in cancer treatment regimens, potentially improving outcomes in vulvar cancer treatment (79). EGCG inhibits several important signaling pathways involved in cancer progression, such as the MAPK pathway, growth factor-related signaling, and NF-κB pathway. These pathways are crucial for cell proliferation and survival, and their inhibition by EGCG can lead to decreased cancer cell growth and increased apoptosis (79). EGCG can modulate epigenetic regulators such as DNA methyltransferases and histone deacetylases and interact with key signaling pathways (e.g., PI3K/Akt/mTOR, MAPK, ERK1/2, p38MAPK) involved in cell proliferation, apoptosis, and autophagy. These actions contribute to the broader anticancer properties of EGCG. However, it can also induce pro-oxidant activity, leading to cytotoxic autophagy and apoptosis in cancer cells (29, 80). EGCG inhibits self-renewal and stem cell markers in cancer stem cells, potentially reducing tumor recurrence and resistance (81). EGCG can enhance the effects of chemotherapeutic agents and reduce their toxicity, but its clinical use is limited due to poor bioavailability. To overcome this, nanotechnology-based delivery systems are being investigated. EGCG from green tea has demonstrated multiple anticancer mechanisms in vulvar and HPV-associated cancers, including degradation of viral oncoproteins, induction of apoptosis, modulation of epigenetic and signaling pathways, and targeting of cancer stem cells. These effects support its potential as a chemopreventive and therapeutic agent, particularly in HPV-induced vulvar cancer, but challenges such as bioavailability remain (81, 82). Although EGCG holds promise in the treatment of vulvar cancer, it is important to consider the variability in response across cancer types and individual patients. The heterogeneity in signaling pathways and cellular responses to EGCG suggests that its efficacy may vary and warrants further investigation to optimize its use in clinical settings. In addition, although EGCG has shown low toxicity and high efficacy in preclinical studies, its effects in human trials are warranted to confirm its safety and efficacy in the treatment of vulvar cancer (81, 82).

## 7 Endometriosis and EGCG relationship

EGCG, exert therapeutic effects over endometriosis mostly through anti-angiogenic, anti-fibrotic, anti-proliferative, and proapoptotic mechanisms.

### 7.1 EGCG mechanisms and pathways in endometriosis

Endometriosis is a chronic gynecological disorder characterized by the growth of endometrial tissue outside the uterus, leading to pain and infertility. Green tea and its major catechin, EGCG, Pro-EGCG, may affect endometriosis through multiple molecular pathways through various biological mechanisms (83–87).

EGCG and Pro-EGCG inhibit the formation of new blood vessels (angiogenesis), which is essential for endometriotic lesion growth. EGCG acts primarily by downregulating VEGF and related pathways, while Pro-EGCG has been shown to downregulate PX domain containing serine/threonine kinase (PXK)-mediated EGF expression by binding to PXK, inhibit angiogenesis via HIF-1α, and inhibit angiogenesis more potently by suppressing EGF-mediated signaling via the HIF-1α/VEGF pathway. This mechanism is crucial during the growth and development of endometriosis, as angiogenesis is an important pathophysiological process in the disease (83, 88). Both compounds reduce lesion size and vascularization in animal models (11, 83, 84). EGCG suppresses the proliferation of endometrial cells and induces apoptosis (programmed cell death) in endometriotic tissue, contributing to lesion regression (11, 28, 29, 84). EGCG and Pro-EGCG regulate immune cell interactions, specifically by reducing dysfunctional macrophages and B cells associated with endometriosis. They target proteins such as metadherin (MTDH) and PXK, alter downstream effectors (e.g., BLK, EGF, MYC, AKT), and reduce immune cell infiltration in lesions (83, 85).

EGCG and Pro-EGCG interact with DNMTs and HDACs, influencing epigenetic modifications that regulate genes involved in angiogenesis and cell proliferation (29, 46, 84, 85). They also demonstrate biological activities that contribute to reduced oxidative stress and inflammation in endometriotic tissue (11, 28, 84, 85). Key proteins are MTDH, PXK, nicotinamide mononucleotide adenylyltransferase 1 (NMNAT1), nicotinamide mononucleotide adenylyltransferase 3 (NMNAT3), and signaling pathways are Akt/PI3K, EGF/Akt1/HIF-1α/VEGF, ERK, p38 MAPK. In addition, immune modulation occurs by down-regulating macrophage and B cell activity and reducing inflammatory gene expression (83, 86).

Pro-EGCG’s inhibition of angiogenesis via HIF-1α is important because this pathway is involved in the response to hypoxia, a condition often found in endometriotic lesions (86, 88). EGCG and Pro-EGCG down-regulate VEGF, a critical factor in angiogenesis. The effects of EGCG on the VEGF pathway are also mediated through the TGF-β1 classical-SMAD pathway and the 67LR-mediated CREB pathway, which are involved in reducing ovarian inflammation and angiogenesis (62). EGCG has been shown to reduce oxidative stress and inflammation via the sirtuin 1 / nod-like receptor protein 3 (SIRT1/NLRP3) pathway, which may contribute to its protective effects against endometriosis (87).

Animal studies consistently show that EGCG and Pro-EGCG reduce lesion size, inhibit angiogenesis, and promote apoptosis in endometriosis models (11, 84). Pro-EGCG shows higher bioavailability and efficacy compared to EGCG, making it a promising candidate for future therapies (11, 85, 86). Neither EGCG nor Pro-EGCG adversely affect ovarian follicles or uterine glands in animal models (11). Most evidence comes from preclinical studies; robust clinical studies are needed to confirm the therapeutic benefits and safety of EGCG and Pro-EGCG in humans (28, 29, 85). Although multiple mechanisms have been identified, further research is needed to fully understand the interaction between immune modulation, angiogenesis, and oxidative stress in the treatment of endometriosis (28, 85, 86).

In conclusion, EGCG and pro-EGCG in green tea inhibit endometriosis progression by targeting angiogenesis, immune cell regulation, apoptosis, and oxidative stress through different molecular pathways. Pro-EGCG provides superior efficacy and bioavailability. These effects are mediated through specific molecular targets and pathways. Most of the evidence comes from preclinical models and clinical validation in humans is needed.

## 8 Polycystic ovary syndrome and EGCG relationship

Polycystic ovary syndrome is a common endocrine disorder in women and is often associated with oxidative stress, inflammation, and metabolic disorders. PCOS is characterized by hormonal imbalances, oxidative stress, and inflammation. The mechanisms by which EGCG exerts its effects in PCOS include modulation of hormonal levels, reduction of oxidative stress, and effects on specific signaling pathways (89–93).

### 8.1 Mechanisms of action and signaling pathways of green tea and EGCG in PCOS

EGCG has been shown to affect hormone levels in PCOS. In human studies, green tea extract (GTE) has been associated with weight loss and a reduction in testosterone levels, which are often elevated in PCOS patients (89, 90). Green tea extract has been shown to decrease testosterone and luteinizing hormone (LH) levels in animal models, while increasing follicle-stimulating hormone (FSH) and progesterone. This hormonal modulation may help restore ovarian function and improve PCOS symptoms (89, 90).

EGCG acts as a potent antioxidant that reduces oxidative stress and inflammation, which are central to PCOS pathology. These effects are mediated through modulation of cellular pathways that control inflammation and oxidative damage (7, 11, 29, 90, 91). It improves mitochondrial function, which may be beneficial in reducing oxidative damage associated with PCOS by strengthening antioxidant defenses (7).

The anti-inflammatory effects of EGCG are mediated through the suppression of inflammatory cytokines such as TNF-a and IL-6. However, these effects have been observed more clearly in animal models (7, 90). EGCG and green tea supplementation may lower fasting insulin, improve insulin resistance, and address underlying metabolic issues in PCOS by reducing body weight and body fat percentage (89–92).

EGCG affects various signaling pathways related to PCOS. It up-regulates the expression of StAR and increases progesterone production in granulosa cells via the 67-kDa laminin receptor-mediated CREB signaling pathway (93). In addition, EGCG inhibits the VEGF pathway, which plays a role in angiogenesis and inflammation, via the TGF-β1 classical-SMAD pathway and the 67LR-mediated CREB pathway. This inhibition can reduce ovarian inflammation and hyperstimulation common in PCOS (62).

### 8.2 Molecular pathways and epigenetic regulation

EGCG interacts with cell surface receptors and intracellular signaling pathways, including MAPK, Akt, and nuclear transcription factors, contributing to its biological effects (7, 29). EGCG may affect gene expression through epigenetic mechanisms and potentially influence the development and progression of PCOS and other gynecological disorders (29). By reducing oxidative stress, EGCG may abolish the effects of increased polyol pathway flux and aldose reductase activity, which play a role in PCOS-associated ovarian dysfunction (94).

EGCG has been shown to improve metabolic parameters in PCOS, for example, it is crucial for managing insulin resistance, which is often seen in PCOS patients (89, 90). The decrease in fasting insulin and free testosterone levels observed in clinical studies suggests that EGCG may be helpful in managing the metabolic and hormonal aspects of PCOS (89). Although EGCG shows promise in managing PCOS through these mechanisms, it is important to consider the complexity of PCOS and the variability in individual responses to treatment. Current evidence, primarily from animal studies and limited human trials, suggests potential benefits, but more extensive clinical studies are needed to fully understand the efficacy and safety of EGCG in managing PCOS. Additionally, EGCG’s role in regulating other pathways, such as the endothelin axis and toll-like receptor (TLR) signaling, highlights its broad biological activity that may have implications beyond PCOS treatment (95).

EGCG are known to enhance ovulation, decrease cyst formation, alleviate generalized hyperalgesia, and reduce plasma corticosterone levels and uterine contractility in dysmenorrhea in humans with PCOS (28). Another study reported potential benefits of GTE supplementation on PCOS. Although human ovarian histology data are lacking animal models have demonstrated GTE’s positive effects on ovarian function. GTE may also improve glycemic control, reduce body weight, LH levels, and androgen concentrations in PCOS (90). Another review reported that EGCG exerts beneficial effects on anthropometric and metabolic parameters, particularly in individuals with PCOS. The results emphasize the therapeutic potential of catechins in PCOS management and support their consideration in clinical treatment strategies (96). EGCG has been shown to significantly reduce pro-inflammatory cytokines such as TNF-*α*, IL-1β and IL-6 in PCOS models, in a dose-dependent manner, suggesting that higher doses may yield greater anti-inflammatory effects In PCOS mouse models, catechins were found out to down-regulate the expression of p-NF-κB p65 and other pro-inflammatory parameters, suggesting a potential mechanism by which catechins reduce uterine inflammation (27). EGCG is known to elevate the activity of antioxidant enzymes like SOD, CAT and GPX in ovarian tissues and help decrease oxidative stress and DNA oxidative damage in PCOS models. Apart from these, catechins are also linked with a reduction in lipid peroxidation markers such as MDA and 8-hydroxy-2′-deoxyguanosine (8-OHdG), emphasizing their role in reducing oxidative damage (97).

In PCOS models, catechins have been shown to improve glucose metabolism and reduce insulin resistance, while also modulating hormonal balance by lowering estradiol, FSH, and LH levels and improving the typically disrupted LH/FSH ratio (27). Another study declared its efficacy in decreasing serum insulin and LH levels and body mass index (BMI) in females with PCOS, showing their potential to target metabolic and hormonal dysregulation. In overweight and obese females with PCOS, green tea consumption has been associated with reductions in body weight and fasting insulin levels (89). In a study, a reduction in BMI and waist circumference was recorded in individuals that daily supplemented with 1,500 mg of GTE for 12 weeks (98). Similarly, a systematic review and meta-analysis reported that women who consumed green tea exhibited lower body weight (99). However, further well-designed clinical trials are needed to validate these findings and establish EGCG as a potential therapeutic agent for PCOS.

Evidence for anti-inflammatory effects in humans is limited, and further research is needed to elucidate the precise molecular mechanisms and long-term benefits (90–92). EGCG may benefit women with PCOS by reducing oxidative stress, improving metabolic and hormonal profiles, and regulating key cellular pathways. While EGCG help regulate inflammation, effective management of PCOS requires a comprehensive approach that addresses its multifaceted endocrine and metabolic nature. Polyphenols, involving EGCG, are being investigated for their potential to target several metabolic and hormonal dysregulations linked with PCOS. However, further research is needed to elucidate the underlying mechanisms and to develop comprehensive treatment guidelines.

Some *in vivo, in vitro*, animal and human studies examining the effects of EGCG on gynecological cancers and diseases are given in Table 4.

**Table 4:** Effects of catechins on gynecologic cancers and diseases: Some *in vivo, in vitro*, animal and human studies.

Gynecological disease	Cell line or model	Dose	Effects	Refs.
	BALB/c mice, 4–5 weeks old	10, 30, or 50 mg/kg EGCG	Upregulation of PTEN expression Downregulation of PDK1, phosphorus (p)-Akt, and p-mTOR expression	(57)
Ovarian cancer	BALB/c nude mice, *n* = 35	10, 30, or 50 mg/kg EGCG per day for 3 weeks	Inhibited tumor growth Upregulated PTEN Downregulated PDK1, p-Akt, and p-mTOR	
	A2780/DDP SKOV-3/DDP	0–40 μM EGCG	Inhibition of cell proliferation and mobility Induction of apoptosis Decreased S100A4 and NF-κB expression Increased p53 expression	(58)
		50 mg/kg EGCG for BALB/c mice (5 weeks old)	Tumor inhibition Ki-67 inhibition p53 upregulation Downregulation of S100A4 and NF-κB expression	
	HeLa cell line, cervical cancer biopsies	ROS production with hydrogen peroxide (0–10 nM), co-cultured with various doses of EGCG	Free radical scavenging with increased SOD and GPX activity Inhibition of cell proliferation	(103)
Cervical cancer	HeLa	EGCG concentration of 60 μg/mL	EGCG was found to be cytostatic but not cytotoxic Cytostatic effect was observed	(104)
	HeLa C33A WI-38	250–500 μM EGCG	Cell-cycle arrest Induction of apoptosis Induction of cell growth	(109)
	HeLa	25–100 g/mL EGCG	Induction of apoptosis Suppression of cell proliferation	(110)
Endometrial cancer				(51)
	RL95-2, AN3-CA Athymic nude mice, *n* = 15	20, 40, or 60 μM pro-EGCG 50 mg/kg of EGCG or pro-EGCG per day for 5 weeks	Activation of p38 MAPK Inhibition of Akt/ERK signaling pathway Inhibition of cellular proliferation Induction of apoptosis Reduced tumor growth Downregulation of NOD1 and NAIP detected	
Endometrial cancer	HeLa SiHa cell line	0–100 μmol/L EGCG Green tea extract (0–250 μg/mL)	Vimentin, ZEB, Slug, Snail, and Twist expression levels decreased There was an increase in E-cadherin expression Effects of TGF-β in HeLa and SiHa cells were reversed Molecular mechanism of TGF-β-induced EMT inhibition was blocked Metastasis was inhibited	(65)
Endometrial cancer	10 case control + 9 cohort studies were examined in a review of the literature (*n* = 19 in total)	Black tea Green tea	Both black tea and green tea protected against endometrial cancer	(49)
Endometrial cancer	Endometrial cancer cells AN3-CA, RL95-2	20–60 μM pro-EGCG; cell proliferation and VEGF-A and HIF-1α signaling pathways were analyzed	PI3K/Akt/mTOR/HIF-1α signaling pathway inhibition Downregulation of CXCL12 restricted the migration and differentiation of macrophages VEGFA secretion reduction Inhibition of infiltration of VEGFA-expressing tumor-associated macrophages	(50)
Vulvar cancer	HFK-HPV18, VIN cl.11	100 μM EGCG	Downregulation of E6 and E7 expression Inhibition of cellular proliferation was detected	(78)
Endometriozis/PCOS	KGN (granulosa-like), hGL (granulosa-lutein), SD rat OHSS model	10–50 μM (*in vitro*), 50 mg/kg (*in vivo*)	Decreased VEGF and TGF-β expression, OHSS progression, and ovarian weight	(111)
	BALB/c female nude mice	8.333 mg/mL EGCG for 16 days	Increased E-cadherin expression Reduced DNA methylation of the E-cadherin promoter region Inhibition of endometrial lesion growth occurred	(112)
Endometriosis	20 C57BL/6 mice	50 mg/kg EGCG or pro-EGCG per day for 21 days	Reduction in endometriotic lesion sizes and overexpression of NMNAT1 and NMNAT3 were detected after pro-EGCG treatment	(85)
	Primary human endometrial stromal cells	0–300 μM pro-EGCG	NMNAT1 and NMNAT3, nicotinamide nucleotide adenylyltransferases increased	(86) (93)
	Human granulosa-like tumor cell line, KGN	0–10 μM EGCG	Human granulosa-like tumor cell line and KGN cells were used Upregulated StAR expression and increased progesterone production were observed	
PCOS	Double blind randomized trial, *n* = 60 30 women were treated and 30 were controls	Intervention group: green tea tablet, 500 mg Control: placebo 12 weeks	Decrease in both free testosterone and insulin levels and weight in the treated group compared to the control group Side effects were observed in the gastrointestinal system	(89)

Akt, Protein kinase B; CXCL12, C-X-C motif chemokine ligand 12; DNA, Deoxyribonucleic acid; EGCG, Epigallocatechin gallate; ERK, Extracellular signal-regulated kinase; GPX, Glutathione peroxidase; HIF-1α, Hypoxia-inducible factor-1alpha; HPV18, Human papillomavirus 18; MAPK, Mitogen-activated protein kinase; mTOR, Mammalian target of rapamycin; NAIP, Neuronal apoptosis inhibitory protein; NF-κB, Nuclear factor kappa-B; NMNAT1, Nicotinamide mononucleotide adenylyltransferase 1; NMNAT3, Nicotinamide mononucleotide adenylyltransferase 3; NOD1, Nucleotide-binding oligomerization domain containing 1; OHSS, Ovarian hyperstimulation syndrome; PCOS, Polycystic ovary syndrome; PDK1, Phosphoinositide-dependent kinase-1; PI3K, Phosphatidylinositol-3 kinase; pro-EGCG, Pro-epigallocatechin gallate; PTEN, Phosphatase and tensin homolog; ROS, Reactive oxygen species; SOD, Superoxide dismutase; StAR, Steroidogenic acute regulatory protein; TGF-β, Transforming growth factor beta; VEGF, Vascular endothelial growth factor; VEGFA, Vascular endothelial growth factor A; ZEB, Zinc finger E-box binding homeobox.

## 9 Safe intake level and toxicity of catechins

While moderate levels of catechin intake from traditional tea consumption are generally considered safe, higher doses, especially from supplements, raise concerns about liver toxicity. Daily EGCG intake from green tea infusions generally ranges from 90 to 300 mg and is considered safe for most people. Even high-level consumers (up to 866 mg per day) rarely experience adverse effects, although rare cases of idiosyncratic liver injury have been reported (100, 101). For concentrated green tea extracts or supplements, a tolerable upper intake level of 300 mg EGCG per day is recommended to provide a safety margin against liver toxicity. Clinical studies show no liver effects below 600 mg/day, but doses of 800 mg or more per day have been associated with increases in liver enzymes, indicating potential harm (100–102). A safe observed level for beverage forms is up to 704 mg EGCG per day, but for solid bolus doses, 338 mg EGCG per day is considered safe (103).

The main risk at high intakes is liver toxicity, especially with supplements or large single doses (100–102). Rare cases of liver injury may occur even at lower intakes due to individual sensitivity. Typical dietary catechin intake from tea and food is much lower than supplemental doses and is not associated with adverse effects. Up to 300 mg/day of EGCG from tea is generally considered safe for healthy adults. This equals about 3–5 cups of brewed green tea per day, depending on strength and variety. One cup of green tea contains 25–86 mg EGCG. EFSA has reported that intakes of >800 mg/day are not safe and present a risk. Excessive consumption can lead to elevated liver enzymes, with the risk being greater when EGCG is taken on an empty stomach or in concentrated supplement form (104).

### 9.1 Limitations

This review is limited by the predominance of observational studies, often relying on food frequency questionnaires and self-reported data, which are subject to recall bias and measurement error. The paucity of high-quality case–control and cohort studies limits causal inference. Significant heterogeneity in study design, methodology, and sample size further complicates cross-study comparisons. Moreover, the direct molecular link between dietary intake and gynecologic disease mechanisms remains insufficiently defined. Robust randomized trials are urgently needed to establish definitive associations and clarify the therapeutic potential of catechins and related compounds in female health.

## 10 Conclusion and future perspective

Catechins, especially EGCG, demonstrate promising therapeutic potential in gynecologic conditions such as endometrial, ovarian, cervical, and vulvar cancers, as well as PCOS and endometriosis. Their effects are mediated through well-characterized mechanisms including antioxidant, anti-inflammatory, antiproliferative, and epigenetic pathways. However, much of the available evidence is preclinical, which limits direct clinical translation. Safety concerns also arise with high-dose or concentrated extract use. Future well-designed human trials are essential to clarify efficacy, optimize dosing, and ensure safety. With appropriate validation, catechins may offer a safe, adjunctive approach to gynecologic care, particularly within personalized and integrative treatment frameworks.

Although some studies report that EGCG exhibits anti-inflammatory, antiproliferative, and anti-angiogenic effects in animal and cell models, these findings remain heterogeneous across gynecologic conditions. Outcomes vary depending on dose, study design, and cell line, and results derived from metastatic models or hormone-dependent pathways are not always consistent. To clarify these conflicting findings, well-designed clinical studies using standardized protocols are needed.

Future research should prioritize well-designed randomized controlled trials and mechanistic studies to clarify the independent effects of catechins and broader dietary patterns on gynecologic disease risk and progression. Current evidence, largely derived from observational studies, remains vulnerable to confounding and cannot establish causality. Integrating dietary factors into gynecologic disease prevention and management demands a multidisciplinary approach that accounts for genetic variability, age, nutritional status, polyphenol intake, environmental exposures, and regional dietary differences. Consideration of factors such as green tea preparation methods, bioavailability, and individual sensitivities (e.g., allergies or hepatotoxicity risk) is essential for developing evidence-based guidelines. Advancing this field will depend on harmonized protocols and diverse population studies to enable the safe and personalized application of dietary strategies particularly catechins as adjuncts in gynecologic care.

## Glossary

8-OHdG: 8-hydroxy-2′-deoxyguanosine
Akt: Protein kinase B
AP-1: Activator protein 1
ATG5: Autophagy related 5
Bcl-2: B-cell lymphoma 2
Bcl-Xl: B-cell lymphoma-extra large
BMI: Body mass index
C: Catechin
CAT: Catalase
CG: Catechin gallate
COX: Cyclooxygenase
CREB: cAMP response element-binding protein
CXCL12: C-X-C motif chemokine ligand 12
DNA: Deoxyribonucleic acid
DNMTs: Dna methyltransferases
EC: Epicatechin
ECG: Epicatechin gallate
ECM: Extracellular matrix
EGC: Epigallocatechin
EGCG: Epigallocatechin gallate
EGF: Epidermal growth factor
EGFR: Epidermal growth factor receptor
EMT: Epithelial mesenchymal transition
ERK1/2: Extracellular signal-regulated kinase
ET-1: Endothelin-1
ET(A)R: Endothelin A receptor
FADD: Fas-associated death domain
FSH: Follicle-stimulating hormone
GC: Gallocatechin
GCG: Gallocatechin gallate
GCL: Glutamate cysteine ligase
GPX: Glutathione peroxidase
GR: Glutathione reductase
GST: Glutathione s-transferase
GTCs: Green tea catechins
GTE: Green tea extract
HDACs: Histone deacetylases
HIF-1α: Hypoxia-inducible factor-1alpha
HK: Glycolytic enzymes hexokinase
HPV16: Human papillomavirus 16
HPV18: Human papillomavirus 18
IARC: International agency for research on cancer
IGF-1: Insulin-like growth factor 1
IL-1β: Interleukin-1β
IL-6: Interleukin-6
JAK/STAT: Janus kinase/signal transducer and activator of transcription
JNK: Jun n-terminal kinases
JUN: Jun proto-oncogene
LH: Luteinizing hormone
MAP: Mitogen-activated protein
MAPK: Mitogen-activated protein kinase
MDA: Malondialdehyde
MMP: Matrix metalloproteinase
MMP-2: Matrix metalloproteinase-2
MMPs: Matrix metalloproteinases
mRNA: Messenger RNA
MTDH: Metadherin
mTOR: Mammalian target of rapamycin
NF-κB: Nuclear factor kappa-B
NFKB1: Nuclear factor kappa B subunit 1
NMNAT1: Nicotinamide mononucleotide adenylyltransferase 1
NMNAT3: Nicotinamide mononucleotide adenylyltransferase 3
Nrf2: Nuclear factor erythroid 2-related factor 2
PCNA: Proliferating cell nuclear antigen
PCOS: Polycystic ovary syndrome
PDK1: Phosphoinositide-dependent kinase-1
PI3K: Phosphatidylinositol-3 kinase
PIK3R: Phosphoinositide-3-kinase regulatory subunit
pro-EGCG: Pro-epigallocatechin gallate
PTEN: Phosphatase and tensin homolog
PXK PX: Domain containing serine/threonine kinase
RNA: Ribonucleic acid
ROS: Reactive oxygen species
RTKs: Receptor tyrosine kinases
SIRT1/NLRP3: Sirtuin 1/Nod-like receptor protein 3
SMAD: Mothers against decapentaplegic
SOD: Superoxide dismutase
StAR: Steroidogenic acute regulatory protein
STAT3: Signal transducers and activators of transcription
TGF-β1: Transforming growth factor beta 1
TLR: Toll-like receptor
TNF-*α*: Tumor necrosis factor-α
ULK1: Unc-51 like kinase 1
VEGF: Vascular endothelial growth factor
VEGFA: Vascular endothelial growth factor A
